# Neonatal propofol exposure impairs synaptic plasticity and cognition, associated with BAG3 upregulation and disrupted synaptic protein homeostasis

**DOI:** 10.3389/fnins.2026.1756936

**Published:** 2026-07-08

**Authors:** Liurong Chen, Chenlu Wang, Yinan Lin, Jie Zhang, Haiying Gao

**Affiliations:** 1 Department of Anesthesiology, The First Affiliated Hospital of Xiamen University, School of Medicine, Xiamen University, Xiamen, China; 2 The School of Clinical Medicine, Fujian Medical University, Fuzhou, China; 3 College of Clinical Medicine, Xiamen Medicine College, Xiamen, China; 4 Department of Anesthesiology, The Second Affiliated Hospital of Fujian Medical University, Quanzhou, Fujian, China; 5 Fujian Provincial Key Laboratory of Neurodegenerative Disease and Aging Research, Institute of Neuroscience, School of Medicine, Xiamen University, Xiamen, Fujian, China; 6 The Key Laboratory of Neural and Vascular Biology, Ministry of Education, Hebei Medical University, Shijiazhuang, Hebei, China; 7 Institute of Neuroscience, Fujian Medical University, Fuzhou, Fujian, China

**Keywords:** BAG3, cognitive function, neonatal mice, neurotoxicity, propofol, synaptic plasticity

## Abstract

**Background:**

Propofol is widely used in pediatric anesthesia, but it has been implicated in adverse effects on brain development following repeated early-life exposure. Bag3, a co-chaperone protein involved in proteostasis and the neuronal stress response, may play a critical role in regulating synaptic function during early brain maturation.

**Methods:**

Neonatal mice were treated with propofol on postnatal days 5–7. Hippocampal neurogenesis was assessed via BrdU immunofluorescence. Synaptic proteins (PSD95, SNAP25) and Bag3 expression were measured by Western blotting. Behavioral performance in adolescence was evaluated using open-field, elevated plus-maze, Morris water maze, Y-maze, and T-maze tests.

**Results:**

Propofol exposure significantly reduced proliferative activity (BrdU incorporation) in the dentate gyrus and decreased PSD95 and SNAP25 expression in both the cortex and hippocampus. Bag3 expression was markedly upregulated, accompanied by a mild increase in its phosphorylated form. Behaviorally, propofol-treated mice showed anxiety-like behavior and impairments in spatial learning and working memory.

**Conclusion:**

These findings suggest that early-life exposure to propofol impairs neurogenesis and synaptic plasticity. This process is temporally associated with the upregulation of stress-responsive co-chaperone BAG3, which precedes the of synaptic protein homeostasis. While causal relationship remains to be established, these findings identifies BAG3 as a candidate correlative marker of anesthetic-induced neurotoxicity and highlights it as a target for future mechanistic studies.

## 1 Introduction

Repeated early life exposure to general anesthetics raises concerns about long-term neurodevelopmental effects. Postnatal exposure to sevoflurane, isoflurane, or propofol impairs neurogenesis, induce synaptic dysfunction, and lead to cognitive deficits in both animal models and human cohorts (Shen et al., 2013; Sun et al., 2016; Warner et al., 2018). The developing brain is particularly vulnerable due to ongoing neurogenesis and synaptic pruning (Feng et al., 2020). A recent transcriptomic study confirms that sevoflurane exposure leads to significant synaptic loss, including PSD95 and synaptophysin (Sun et al., 2025), highlights disrupted synaptic integrity as a reproducible andconsequence of early anesthesia.

Propofol is a widely used in pediatric procedures but repeated neonatal exposure is linked to long-term synaptic and cognitive deficits (DiMaggio et al., 2009). Proposed mechanisms include calcium dysregulation, mitochondrial dysfunction, and autophagic imbalance (Wilson, 1995; Hall et al., 2012), yet the molecular mediators linking propofol to persistent synaptic alterations remain incompletely understood (Wilson, 2003). Disruptions in synaptic protein homeostasis may underlie these deficits. In this context, Bcl-2-associated athanogene 3 (Bag3), a co-chaperone protein involved in proteostasis and stress responses (Gamerdinger et al., 2009), has emerged as a potential regulator of synaptic integrity. Bag3 facilitates synaptic proteins t turnover via Hsp70 and the cytoskeleton, and its dysregulation under stress could contribute to synaptic dysfunction (Behl, 2016; Lin et al., 2022).

Postsynaptic density protein 95 (PSD95) and synaptosomal-associated protein 25 (SNAP25) are established markers of synaptic plasticity. PSD95 scaffolds excitatory postsynaptic receptors, while SNAP25 mediates vesicle fusion. Reduced expression of these proteins correlates with impaired synaptic strength and cognition in neurotoxicity models (Editorial, 1976; Corradini et al., 2009), making them useful indicators of anesthetic-induced synaptic disruption.

Bcl-2-associated athanogene 3 (Bag3) is a stress-responsive co-chaperone that maintains proteostasis by selective autophagy and apoptosis (Gamerdinger et al., 2009; Behl, 2011). It interacts with heat shock protein 70 family (Hsp70) to clear misfolded proteins. In the nervous, Bag3 supports synaptic maintenance, dendritic integrity, and survival (Santoro et al., 2017; Meister-Broekema et al., 2018), positions it as a potential regulator of developmental synaptic integrity (Lin et al., 2022).

Metabolic and proteotoxic stress are established consequences of general anesthetics. Given that propofol impairs mitochondrial function and calcium homeostasis, we hypothesized that these insults may trigger Bag3 upregulation as a compensatory stress response. Although Bag3 is well studied in cardiomyopathy and cancer, its role in anesthetic-induced developmental synaptic disruption is unexplored. Its capacity to regulate turnover of synaptic suggests dysregulation could link initial anesthetic stress to persistent synaptic protein dyshomeostasis.

Previous studies show neonatal propofol induces apoptosis, oxidative stress, and mitochondrial dysfunction (Jevtovic-Todorovic et al., 2003; Yon et al., 2005), but the pathways connecting initial insult to long-term deficits are unclear. We focused on BAG3 as a potential mechanistic link. We hypothesize that propofol elicits a metabolic/proteotoxic stress marked by acute BAG3 upregulation, which disrupts homeostasis of c PSD95 and SNAP25, impairing synaptic plasticity and contributing to cognitive deficits. Clinical evidence remains inconsistent: the GAS trial found no significant neurodevelopmental effects after single brief exposure (Sun et al., 2016), while other cohorts link multiple exposures to behavioral problems (Warner et al., 2018; Feng et al., 2020), highlighting the need for precise molecular insights.

Given Bag3’s role in proteostasis and synaptic regulation, we hypothesize that neonatal propofol disrupts hippocampal cell proliferation and impairs synaptic plasticity/cognitive through a Bag3-dependent mechanism. We assessed Bag3 expression immediately after exposure and synaptic protein integrity at a later stage. We further hypothesized that post-insult metabolic modulator lonidamine could attenuate these deficits.

Several lines of evidence prioritize BAG3 in anesthetic-induced synaptic disruption. First, BAG3 sits at the intersection of proteostasis, autophagy, and synaptic integrity, mechanisms implicated in anesthetic neurotoxicity. BAG3 functions as an autophagy adaptor in neurons, facilitating the clearance of aggregation-prone proteins and maintaining synaptic homeostasis through Hsp70 and the postsynaptic cytoskeleton (Ji et al., 2019; Lin et al., 2022). Second, transcriptomic analyses identify BAG3 as a stress-responsive and altered by anesthetic in the developing brain (Song et al., 2019; Yu, 2021). Third, unlike general stress markers (e.g., HSP70, caspase-3), BAG3’s dual role in protein quality control and synapse-specific regulation makes it a compelling candidate bridging global stress and localized synaptic dysfunction. While other pathways are extensively studied (Jevtovic-Todorovic et al., 2003; Yon et al., 2005), BAG3 remains underexplored in anesthetic neurotoxicity,

Among stress-responsive proteins, BAG3 was selected for its synapse-specific functions and role in selective autophagy. We propose a working hypothesis: neonatal propofol → acute metabolic/proteotoxic stress → rapid BAG3 upregulation → potentially excessive autophagic turnover of synaptic scaffolding proteins proteins → persistent synaptic protein dyshomeostasis → impaired synaptic plasticity → long-term cognitive/emotional deficits. This model is hypothesis-generating; causal validation remains direct genetic manipulation.

## 2 Materials and methods

### 2.1 Animals and grouping

Both sexes of Wild-type (WT) C57BL/6 neonatal mice were obtained from the Laboratory Animal Center of Xiamen University. All procedures were approved by the Institutional Animal Care and Use Committee and conducted in accordance with the National Institutes of Health Guide for the Care and Use of Laboratory Animals. A total of 12 neonatal mice from 3 litters were used in this study. Postnatal day 5 (P5) pups were randomly assigned to two groups: the propofol group (*n* = 6) received intraperitoneal injections of propofol (50 mg/kg) three times at 2-h intervals for three consecutive days (P5-P7), and the control group (*n* = 6) received an equivalent volume of intralipid (vehicle) on the same schedule. The sample size of *n* = 6 per group was determined based on previous studies in the field (Milanovic et al., 2017) and was sufficient to achieve statistical power. All 12 animals (100% survival rate) successfully tolerated the anesthetic and recovery periods and survived until the predetermined endpoints for tissue collection. No animals were excluded or replaced during the course of the experiment, as all subjects met the pre-established health criteria for inclusion, which included normal body temperature, successful recovery from anesthesia following each injection, and the absence of any signs of distress or physical injury. Mice were housed in standard cages under a 12-h light/dark cycle with ad libitum access to food and water.

### 2.2 Drug administration and body weight monitoring

Propofol (Diprivan) (AstraZeneca, Cambridge, UK) was diluted in 10% intralipid to a final concentration of 10 mg/mL. Body weight was recorded daily from P5 to P30 to monitor developmental effects associated with propofol exposure. Propofol (Diprivan) (AstraZeneca, Cambridge, UK) was diluted in 10% intralipid to a final concentration of 10 mg/mL. Neonatal mice received intraperitoneal injections of propofol (50 mg/kg) three times at 2-h intervals on P5–P7. This dosing regimen was selected based on established protocols that effectively induce deep anesthesia and model repeated neonatal exposure in rodents, leading to robust and reproducible neurodevelopmental effects (Gonzales et al., 2015; Milanovic et al., 2017). Body weight was recorded daily from P5 to P30 to monitor developmental effects associated with propofol exposure.

### 2.3 Experimental timeline, physiological management, and rationale

The selection of all key time points in this study was strategically designed to capture the progression of events, from the immediate molecular stress response to the long-term synaptic and functional consequences of neonatal propofol exposure. To ensure that the observed effects were attributable to the direct neurotoxic actions of propofol rather than secondary systemic compromise, stringent physiological support was maintained throughout the anesthetic period. All pups were kept on a thermostatically controlled heating pad to maintain core body temperature at approximately 37 °C and were provided with humidified 50% oxygen at a flow rate of 1 L/min. This supportive care protocol is established in neonatal rodent anesthesia models to maintain physiological homeostasis (Milanovic et al., 2017). Propofol Exposure (P5–P7): This period corresponds to the brain growth spurt in rodents, a phase of intense synaptogenesis and gliogenesis that is highly vulnerable to external insults (Semple et al., 2013). Anesthetic exposure during this conserved developmental window enables modeling of the potential neurodevelopmental risks associated with early-life anesthesia in humans.

#### 2.3.1 Acute molecular analysis (P7/P8)

Brain tissues were collected immediately after the final propofol exposure (P7 for Western blot) or after a short recovery period (P8 for immunofluorescence). This time point captures the acute BAG3 stress response, chosen specifically to capture the primary molecular stress response triggered by propofol, prior to the manifestation of overt structural damage. Assessing the expression of the stress-responsive co-chaperone Bag3 at this stage allows us to identify it as a potential initiating factor in the subsequent pathological cascade.

#### 2.3.2 Synaptic protein assessment (P21)

Synaptic protein expression (e.g., PSD95, SNAP25) was analyzed at postnatal day 21. This time point captures the state of synaptic protein homeostasis and corresponds to a later developmental stage in mice, coinciding with the peak of synaptogenesis and active circuit refinement (Zuo et al., 2005). Investigating synaptic alterations at P21 enables us to determine whether the early-life propofol insult leads to persistent disruptions in synaptic protein homeostasis, which may underlie the observed long-term behavioral deficits.

#### 2.3.3 Behavioral testing (P35–P37)

Behavioral assessments were conducted during adolescence (starting from P35). This time point captures the long-term functional outcomes and is critical for distinguishing the long-term functional outcomes from the acute pharmacological effects of the drug. Evaluating emotional and cognitive functions at this stage enables the detection of persistent neurodevelopmental impairments resulting from the neonatal intervention.

### 2.4 Immunofluorescence labeling (BrdU, GluR1, synaptophysin)

To assess the acute impact of propofol on cellular proliferation, neonatal mice received a single high-dose intraperitoneal injection of BrdU (500 mg/kg, Sigma-Aldrich, B5002) 2 h prior to sacrifice on P8. This short labeling interval was strategically chosen to capture the population of cells undergoing S-phase DNA synthesis during the immediate recovery period from the final propofol exposure. This approach minimizes the confounding effects of long-term cell survival and differentiation, thereby providing a more direct measure of the anesthetic’s effect on the proliferative activity itself (Kempermann et al., 1997). Any potential nonspecific effects of the high-dose BrdU regimen were controlled for, as both the experimental and control groups received identical injections.

Whole mouse brain tissues from individual mice were fixed in 4% paraformaldehyde (PFA) and embedded with 4% agarose. The brain coronal sections (thickness, 50 μm) were incubated in PBS overnight with horizontal shaking in a 6-well plate. After washing with distilled water, the sections were treated with 2 mol/L hydrochloric acid for 30 min at 37 °C to denature DNA, then rinsed in 0.1 mol/L boric acid buffer (pH 8.5) for neutralization. The sections were incubated with the mixed primary antibodies against BrdU (1,500, Abcam, ab6326) at 4 °C overnight, washed, and incubated with the mixed fluorescent secondary antibodies (1,1,000, Thermo Fisher) at room temperature for 4 h in the dark, followed by nuclear staining with DAPI (Beyotime, Shanghai, China; Cat# C1005). All sections were carefully inspected for physical integrity before imaging. Sections with tears, folds, or uneven background were excluded from analysis. The sections were mounted and examined using a Zeiss LSM 880 confocal microscope.

To comprehensively assess proliferative activity, two complementary quantification approaches were employed. First, due to the short 2-h BrdU labeling interval, which resulted in a sparse population of labeled cells, we quantified the integrated fluorescence intensity of the BrdU signal within the dentate gyrus (DG) as a measure of overall DNA synthesis activity. The region of interest (DG) was outlined based on DAPI staining, and the mean fluorescence intensity was measured. Background subtraction was performed uniformly across all images. Second, to validate these findings and align with conventional field reporting standards, BrdU^+^ cells were manually counted by two blinded observers. Cells were identified based on clear co-localization of BrdU signal with DAPI-stained nuclei. Cell densities were calculated as the number of BrdU^+^ cells per mm^2^ of the DG area. Both quantification methods yielded consistent results. This study did not evaluate earlier progenitor cell stages, as markers such as Ki67, Sox2, or Tbr2 were not assessed.

### 2.5 Western blotting

The cortex and hippocampus were dissected separately on ice and homogenized in RIPA lysis buffer supplemented with protease and phosphatase inhibitors. Protein concentrations were determined with a BCA assay (BioRad; Hercules, CA, USA). Equal amounts of protein (20–40 μg per lane) were separated by SDS-PAGE and transferred onto PVDF membranes (Millipore; Bedford, MA, USA). Membranes were blocked with 5% skim milk and incubated overnight at 4 °C with the following primary antibodies: anti-PSD95 (1:1000; Abcam Cambridge, UK; ab18258), anti-SNAP25 (1:1000; Cell Signaling Technology, Danvers, MA, USA; #11111), anti-GluR1 (1:1000, Proteintech, Rosemont, IL, USA; Cat#67642-1-Ig), anti-Bag3 (1:1000; Abcam, ab47124), anti-phospho-Bag3 (1:500; Bioss (Woburn, MA, USA), bs-5519R), anti-GAPDH (1,5,000; Proteintech; 60,004-1-Ig), and anti-*β*-actin (1,3,000, Affinity, Jiangsu, China; AF7018). After washing, membranes were incubated with HRP-conjugated secondary antibodies (1,5,000; Abcam) for 1 h at room temperature. Protein bands were visualized with enhanced chemiluminescence (Thermo Fisher Scientific, Waltham, MA, USA) and imaged using a ChemiDoc MP system (Bio-Rad). Band intensities were quantified in ImageJ and normalized to GAPDH.

The choice of Western blotting for PSD95 and SNAP25 at P21 was driven by the need for quantitative, region-specific total protein analysis at a later developmental stage. Immunofluorescence for GluR1 and synaptophysin at P8 was chosen to capture both expression levels and subcellular localization patterns immediately after anesthetic exposure, allowing detection of regional and compartment-specific changes.

### 2.6 Behavioral tests

All behavioral assessments were conducted during the light phase by trained experimenters who were blinded to the group assignment. Videos were recorded and analyzed with ANY-maze software (Stoelting; Wood Dale, IL, USA).

#### 2.6.1 Open field test (OFT)

General locomotor activity and anxiety-related behavior were evaluated using the OFT, as described previously (Seibenhener and Wooten, 2015). The open-field arena (50 × 50 × 42 cm) was divided into central and peripheral zones. Mice were placed individually in the center and allowed to explore for 5 min. The total distance traveled and time spent at the center were recorded.

#### 2.6.2 Elevated plus maze (EPM)

The plus-shaped apparatus consisted of two open arms (30 × 5 cm) and two closed arms (30 × 5 × 15 cm) elevated 50 cm above the floor. Mice were placed on the central platform and allowed to explore for 10 min. Time spent in the open arms and the number of open-arm entries were recorded.

#### 2.6.3 Morris water maze (MWM) test

Spatial learning was assessed using the Morris water maze (MWM), as described previously (Zhuang et al., 2018). The circular pool (120 cm in diameter) was filled with opaque water maintained at 22–24 °C. A hidden platform (10 cm in diameter) was positioned 1 cm below the water surface. Mice underwent 5 days of training (four trials per day), and the latency to reach the platform was recorded. A probe test was conducted on day 6, during which the platform was removed and the time spent in the target quadrant was measured.

#### 2.6.4 T-maze and Y-maze

For the Y-maze, spontaneous alternation was assessed during a 5-min exploration period. The alternation rate was calculated as: alternation (%) = [number of triads with entries into all three arms/(total arm entries − 2)] × 100.

In the T-maze, spatial working memory was evaluated by training mice to locate a food reward placed in one arm. The percentage of correct choices was calculated over five trials per day.

### 2.7 Pharmacological intervention with lonidamine

To investigate the potential reversibility of propofol-induced deficits after their establishment, a subset of propofol-exposed mice received pharmacological intervention with lonidamine during adolescence. Lonidamine was selected as a proof-of-concept metabolic modulator because it inhibits glycolysis and mitochondrial pyruvate transport, thereby modulating cellular energy metabolism and stress signaling (Fantin et al., 2006; Pelicano et al., 2006). This choice was grounded in our hypothesis that propofol-induced metabolic stress is an upstream driver of BAG3 upregulation and subsequent synaptic protein loss. Lonidamine is not intended as a clinically ready therapeutic but rather as a tool to probe the metabolic stress component of the proposed cascade. Beginning at postnatal day 30 (P30), these mice were randomized to receive either lonidamine (20 mg/kg, i.v., *n* = 6) or an equivalent volume of vehicle (*n* = 6) by intravenous injection every other day until P37. The lonidamine dose (20 mg/kg) was selected based on established protocols in rodent models demonstrating effective modulation of neuronal metabolism and mitigation of stress-induced dysfunction without overt toxicity (Pelicano et al., 2006; Wang et al., 2025). The treatment window (P30–P37) was chosen to commence after the emergence of synaptic protein deficits (observed at P21), but prior to the completion of adolescent brain maturation, to evaluate the reversibility of established deficits during a potential therapeutic window. The selection of the P30-P37 window for intervention was based on the following rationale: it follows the manifestation of synaptic protein deficits (observed at P21) but precedes the completion of adolescent brain maturation, thereby representing a potential therapeutic window for mitigating established functional impairments. It is important to note that an additional control group (i.e., vehicle-treated animals that had not been exposed to propofol) was not included in this intervention paradigm. The primary aim of this experiment was to test whether lonidamine could reverse deficits specifically in propofol-exposed animals, rather than to characterize the baseline effects of lonidamine in healthy adolescents. Future studies will include this additional control group to fully dissect the compound’s effects.

### 2.8 Data analysis

All statistical analyses were performed with GraphPad Prism 9.0 (GraphPad Software, USA). Data are expressed as mean ± SEM. Comparisons between two groups were conducted using an unpaired two-tailed Student’s t-test. For multiple comparisons, a one-way ANOVA followed by the least significant difference (LSD) *post hoc* test was applied. *p* < 0.05 was considered statistically significant.

The sample size of *n* = 6 per group for all experiments (molecular, histological, and behavioral) was determined based on established protocols in the field of developmental anesthetic neurotoxicity (Amrock et al., 2015; Milanovic et al., 2017). This sample size has been consistently demonstrated to provide sufficient statistical power to detect large effect sizes characteristic of the robust neurodevelopmental perturbations induced by neonatal anesthetic exposure. Furthermore, the effect sizes observed in our key outcome measures (e.g., Cohen’s d > X for behavioral deficits) confirm that the study was adequately powered to test our primary hypotheses.

## 3 Results

### 3.1 Propofol acutely suppresses neuronal proliferative activity in the dentate gyrus (DG)

**Figure 1:**
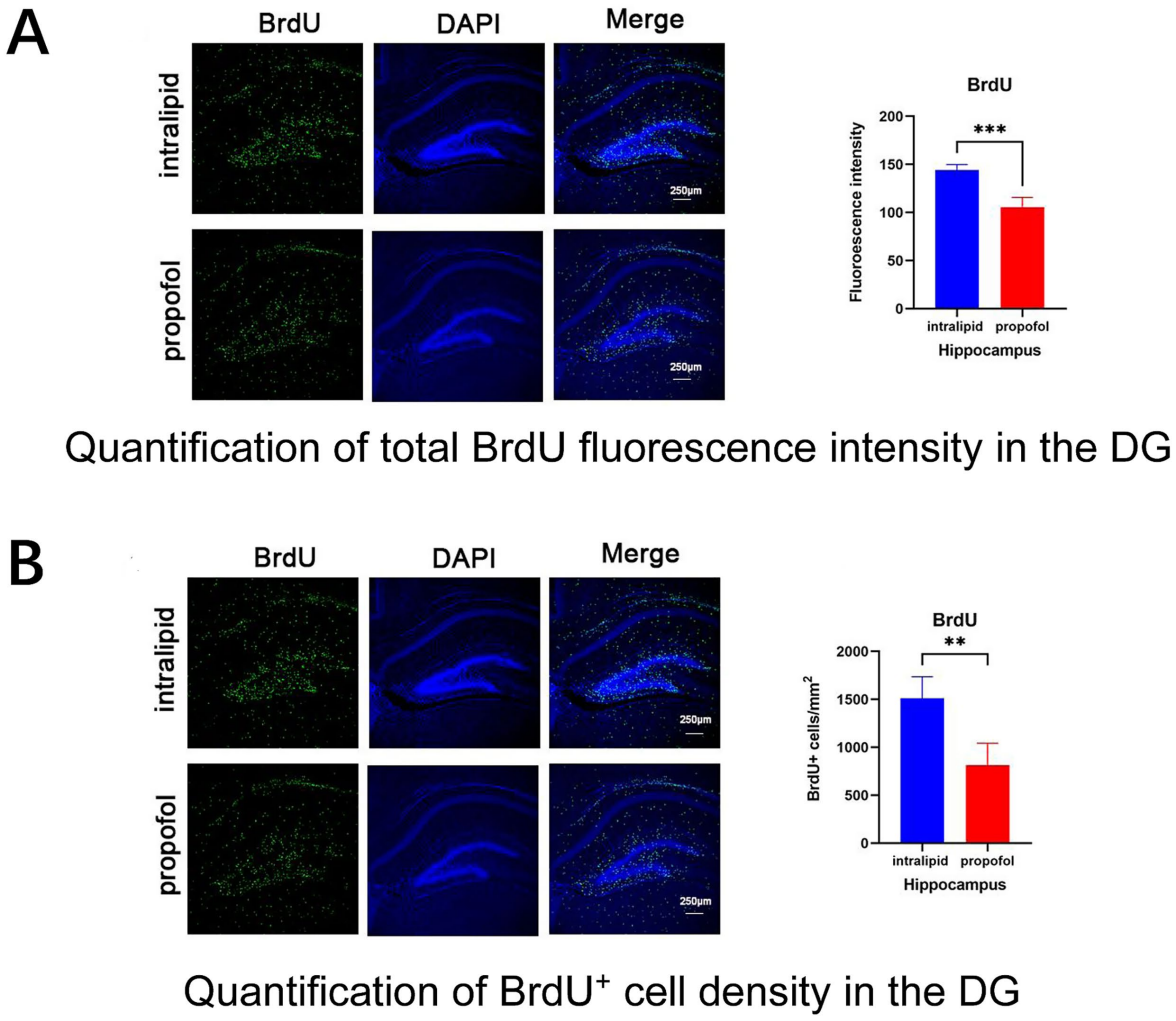
Neonatal propofol exposure acutely suppresses proliferative activity in the dentate gyrus. **(A)** Representative confocal images (left) and quantification (right) of BrdU fluorescence intensity in the dentate gyrus (DG) at postnatal day 8 (P8). BrdU^+^ cells (green) are shown co-localized with DAPI (blue). Due to the short 2-h BrdU labeling window, fluorescence intensity provides an unbiased measure of cumulative DNA synthesis activity. Scale bar = 250 μm. Propofol exposure significantly reduced BrdU fluorescence intensity compared to controls. **(B)** Representative confocal images (left) and quantification (right) of BrdU^+^ cell density in the DG at P8. BrdU^+^ cells (green) were identified based on clear co-localization with DAPI-stained nuclei (blue) and counted manually by two blinded observers. Scale bar = 250 μm. The cell-counting results are consistent with the fluorescence intensity data, further confirming the suppressive effect of propofol on hippocampal proliferation. The difference in SYN staining patterns between hippocampus **(A)** and cortex **(B)** reflects known regional heterogeneity in synaptic organization. Data are represented as mean ± SEM. *n* = 5–6 per group, **p < 0.01 and ***p < 0.001 vs. control group.

To investigate the acute impact of repeated propofol exposure on cell proliferation during early development, neonatal mice received intraperitoneal injections of propofol (50 mg/kg) three times daily from postnatal day 5 (P5) to P7. Two hours prior to sacrifice on P8, the mice were administered a single high-dose injection of BrdU (500 mg/kg) to label dividing cells. Brain tissues were collected, and coronal sections of the hippocampus were processed for immunofluorescence staining with anti-BrdU antibody and counterstained with DAPI to visualize cell nuclei. Representative images of the dentate gyrus (DG) are shown in Figure 1A, clearly demonstrating the punctate nuclear morphology of BrdU^+^ cells and their co-localization with DAPI. Because we used a short 2-h BrdU pulse to capture acute proliferation, the number of labeled cells was relatively sparse, making traditional cell counting potentially subjective. Therefore, to obtain an objective, unbiased measurement of overall proliferative activity under these conditions, we first quantified the total BrdU fluorescence intensity within the DG region (Figure 1A). This approach provides an unbiased measure of cumulative DNA synthesis activity. As shown in Figure 1A, propofol-treated mice displayed a significant reduction in BrdU fluorescence intensity in the DG compared with controls at this early time point, indicating a marked suppression of hippocampal proliferative activity induced by neonatal propofol exposure. To ensure the robustness of our findings and to align with conventional field reporting standards, we also performed manual counting of BrdU^+^ cells by two blinded observers (Figure 1B). Although cell counting can be more subjective with sparse labeling, the results were consistent with the fluorescence intensity data, showing a significant reduction in BrdU^+^ cell density in the propofol-treated group.

These complementary analyses further confirm the inhibitory effect of propofol on neonatal hippocampal proliferation. Taken together, these results indicate that repeated neonatal exposure to propofol markedly suppresses proliferative activity in the hippocampal DG shortly after exposure.

### 3.2 Propofol decreases synaptic protein expression

To determine whether repeated neonatal exposure to propofol disrupts synaptic protein development, we examined the expression levels of two key synaptic markers- PSD95 and SNAP25-in the hippocampus and cortex of mice at P21. These proteins are crucial for maintaining synaptic structure and neurotransmitter release, and are frequently used to assess synaptic maturation and integrity. This time point was selected to capture persistent alterations in synaptic protein expression following propofol administration during the early postnatal period (P5–P7).

**Figure 2:**
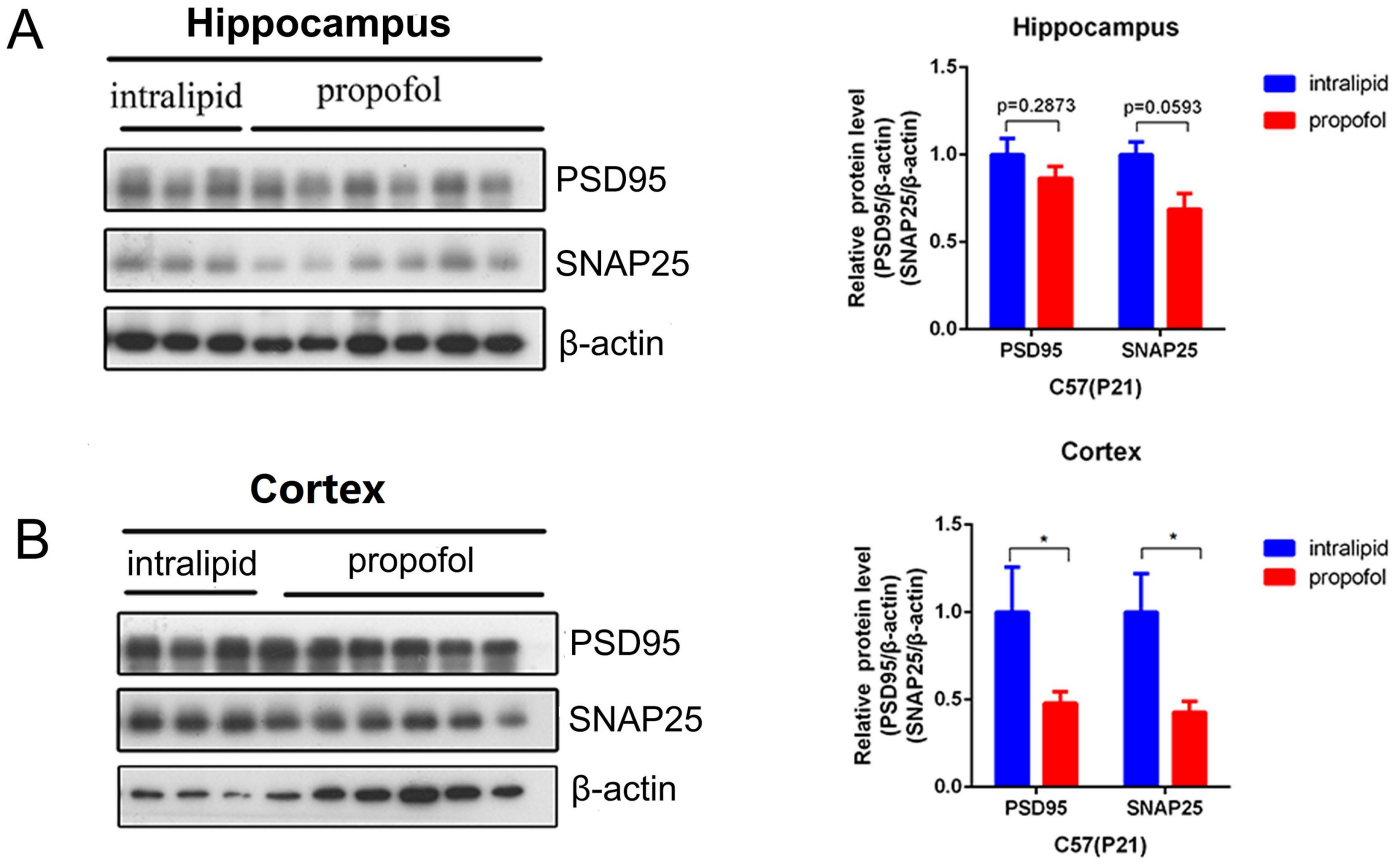
Repeated neonatal propofol exposure decreases synaptic protein expression in the hippocampus and cortex. Postnatal C57BL/6 mice were treated with propofol (50 mg/kg, intraperitoneal, 3×/day) from postnatal day 5 (P5) to P7. Brain tissues were collected at P21 to evaluate the long-term impact on synaptic protein development. Western blotting was used to detect PSD95 and SNAP25, two markers of synaptic structure and neurotransmitter release. **(A,B)** Left: Representative Western blot images of PSD95 and SNAP25 expression in hippocampal **(A)** and cortex **(B)** lysates from control (intralipid) and propofol-treated mice. Right: Densitometric quantification of PSD95 and SNAP25 protein levels normalized to *β*-actin in the hippocampus **(A)** and cortex **(B)**. Quantification of BrdU fluorescence intensity in the DG. Data are presented as mean ± SEM; *n* = 3–6 mice per group; *p* < 0.05, one-way ANOVA with LSD *post hoc* test. PSD95- postsynaptic density protein 95; SNAP25-synaptosomal-associated protein 25; β-actin-glyceraldehyde-3-phosphate dehydrogenase.

As shown in Figure 2A, Western blot analysis of hippocampal lysates revealed that both PSD95 and SNAP25 levels were modestly reduced in the propofol-treated group compared with the control group. In contrast, more pronounced reductions in both proteins were observed in cortical samples (*p* < 0.05; Figure 2B), suggesting that the cortex may be more sensitive to propofol-induced synaptic disruption. These results suggest that repeated exposure to propofol during a critical postnatal period disrupts synaptic protein accumulation, particularly in cortical regions, which may contribute to downstream deficits in neural connectivity and cognitive function.

### 3.3 Altered expression of synaptic markers GluR1 and synapsin following neonatal propofol exposure

To further investigate the impact of neonatal propofol exposure on synaptic development, we examined the expression of GluR1, a key excitatory postsynaptic receptor, and SYN, a presynaptic vesicle-associated protein involved in neurotransmitter release.

**Figure 3:**
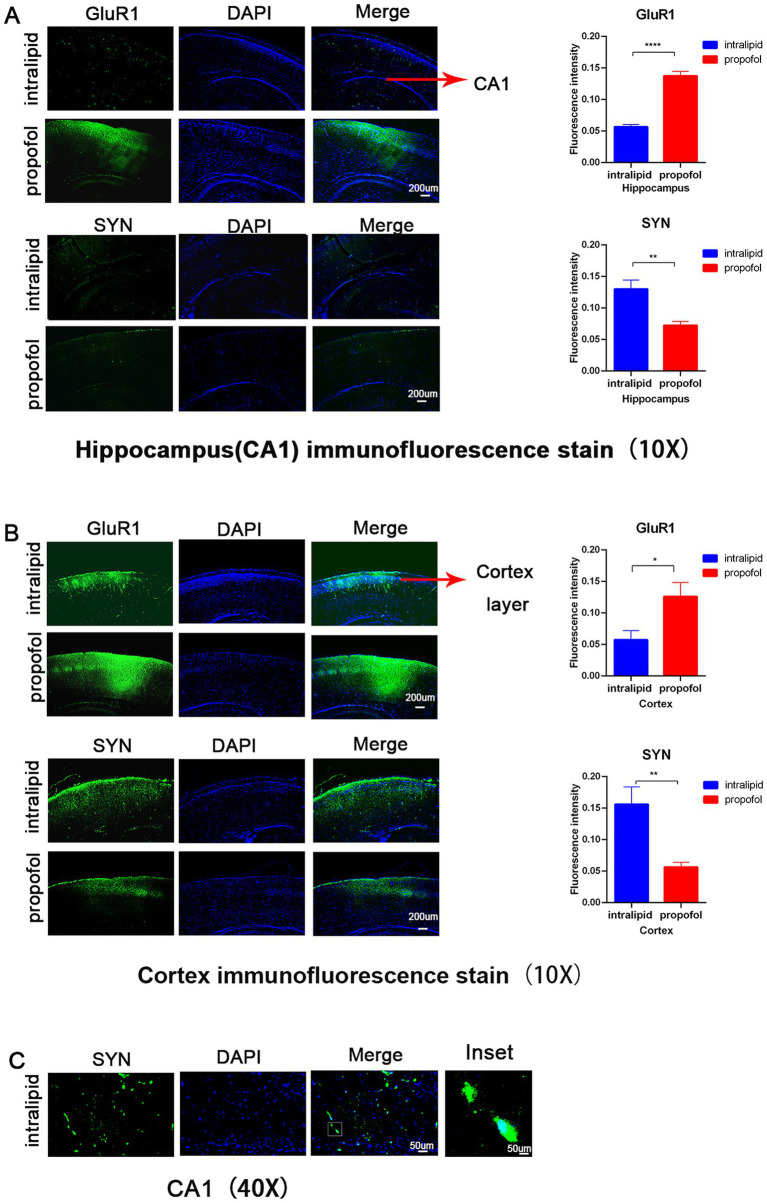
Neonatal propofol exposure leads to divergent changes in GluR1 and synaptophysin expression. Immunofluorescence staining for the postsynaptic AMPA receptor subunit GluR1 and the presynaptic vesicle protein synaptophysin (SYN) was performed on brain sections collected at P8. DAPI was used as a nuclear counterstain. **(A)** Representative images of the hippocampal CA1 region. Propofol exposure resulted in a marked increase in GluR1 immunofluorescence intensity, while SYN signal was substantially decreased compared to controls. The regions of interest are indicated (CA1). Scale bar = 200 μm, original magnification100X. Quantification of mean fluorescence intensity is shown on the right. **(B)** Representative images of the cortical region. Consistent with the hippocampus, GluR1 signal was increased and SYN signal was decreased in the cortex of propofol-treated mice. Cortical layers are indicated for orientation. Scale bar = 200 μm, original magnification100X. **(C)** High-magnification representative image of SYN staining from a control hippocampal section, showing specific punctate labeling and low background. Scale bar = 50 μm, original magnification400X. Quantifications of mean fluorescence intensity for GluR1 and SYN are shown on the right of **A** and **B**. Data are presented as mean ± SEM; *n* = 5–6 per group; *p < 0.05, **p < 0.01, ****p < 0.0001 *vs*. control group.

Immunofluorescence analysis revealed significant increases in GluR1 expression in both the hippocampus and cortex of propofol-treated mice compared with controls (Figures 3A,B). In contrast, SYN expression was markedly decreased in both regions. Quantitative analysis showed that GluR1 levels were significantly elevated in the hippocampus (*p* < 0.001) and cortex (*p* < 0.05), whereas SYN expression was significantly reduced in the hippocampus (*p* < 0.001) and cortex (*p* < 0.01) (Figures 3A,B). These immunofluorescence findings demonstrate a clear divergence in the expression of pre- and postsynaptic markers following propofol exposure. Collectively, these changes suggest an imbalance between excitatory receptor availability and presynaptic signaling capacity, potentially reflecting dysregulated synaptic maturation. Taken together, the divergent expression patterns of GluR1 and SYN imply that neonatal exposure to propofol alters both pre- and postsynaptic compartments, favoring enhanced excitatory receptor signaling in the absence of adequate presynaptic vesicle organization. Importantly, the distinct SYN staining patterns observed in the hippocampus versus the cortex are consistent with known regional differences in synaptic architecture, and no overt section damage was observed in the analyzed tissues (exemplified by a high-magnification image from an intact control section, Figure 3C).

Our findings of persistent synaptic protein alterations are strongly supported by a recent transcriptomic study. Sun et al. (2025) demonstrated that early-life exposure to sevoflurane, another commonly used general anesthetic, led to significant downregulation of key synaptic markers, including PSD95 and synaptophysin, in the offspring, and identified associated disruptions in critical neurodevelopmental pathways (Sun et al., 2025). This convergence of evidence across different anesthetics and experimental approaches underscores that disruption of synaptic integrity is a common and enduring consequence of early anesthetic exposure. This uncoordinated synaptic remodeling may contribute to the long-term cognitive and behavioral abnormalities observed in later developmental stages.

It should be noted that the analytical methods for synaptic markers were selected based on distinct experimental objectives: Western blotting for quantitative total protein assessment (PSD95, SNAP25 at P21) and immunofluorescence for spatially resolved distribution analysis (GluR1, synaptophysin at P8). Despite these methodological differences, the findings consistently support propofol-induced disruption of synaptic protein homeostasis.

### 3.4 Propofol increases Bag3 expression

Given its proposed role in synaptic protein turnover and neurodevelopmental regulation, Bag3 was selected as a candidate mediator of propofol-induced synaptic dysfunction. Bag3 is a co-chaperone protein involved in proteostasis, autophagy, and neuronal stress responses and has recently been implicated in the maintenance of synaptic structure (Behl, 2011; Santoro et al., 2017; Zhou et al., 2020).

**Figure 4:**
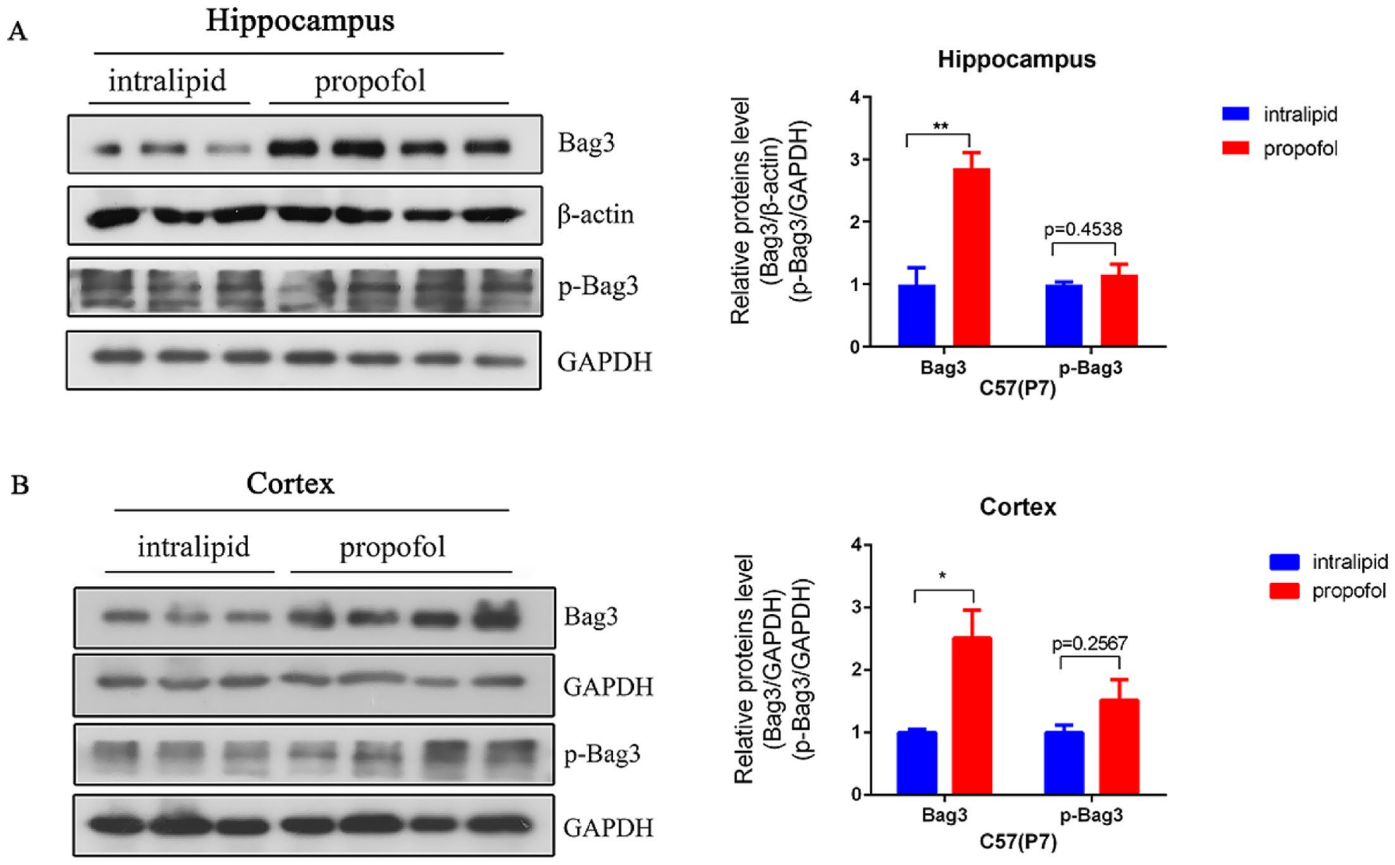
Propofol induces early upregulation of Bag3 expression and phosphorylation. **(A)** Representative Western blots and quantification of Bag3 and p-Bag3 expression in the hippocampus. Protein levels were normalized to β-actin. **(B)** Representative Western blots and quantification of Bag3 and p-Bag3 expression in the cortex. Protein levels were normalized to GAPDH. Data are shown as mean ± SEM; *n* = 3–6, *p* < 0.01–0.001. *p < 0.05, **p < 0.01 vs. control group.

To investigate whether propofol exposure triggers early molecular stress responses that may precede synaptic alterations, we examined the expression of Bag3 in the hippocampus and cortex immediately after propofol treatment (P7). Western blot analysis of hippocampal and cortical lysates collected at postnatal day 7 (P7), immediately after the final propofol exposure, revealed a substantial increase in Bag3 protein levels in both regions. Bag3 was measured at this time point to capture early molecular responses to anesthetic exposure and to evaluate its potential role as an initiating factor in synaptic remodeling. Representative immunoblots illustrate enhanced Bag3 expression in the propofol-treated group (Figure 4A). Similarly, a substantial increase in Bag3 protein levels was also observed in the cortex of propofol-treated mice (Figure 4B). This early BAG3 upregulation at P7 temporally precedes the loss of PSD95 and SNAP25 observed at P21 (Figure 2), supporting its potential role as an upstream mediator of propofol-induced synaptic protein dyshomeostasis. We also assessed the phosphorylation status of Bag3, a post-translational modification associated with functional regulation, and observed a mild, non-significant increase in both brain regions. These findings suggest that neonatal propofol exposure activates Bag3 expression as part of an early stress response. The temporal association (BAG3 upregulation at P7 preceding synaptic protein loss at P21) is consistent with a potential role in downstream synaptic remodeling, but causal inference requires direct experimental validation a key question arising from our findings is what triggers this rapid upregulation of Bag3. While traditionally viewed as a response to proteotoxic stress, emerging evidence indicates that metabolic disturbance is a critical upstream event. In a recent comprehensive review, Fei et al. (2025) delineate how anesthetics, including propofol, can disrupt cerebral glucose homeostasis and enhance glycolytic flux, creating a state of metabolic stress that precedes neuronal dysfunction. These findings, along with reports implicating anesthetic exposure in cerebral metabolic stress (Fei et al., 2025), support the hypothesis that the observed Bag3 upregulation may represent a direct response to propofol-induced metabolic imbalance. If validated, Bag3 could serve as a molecular nexus linking the initial anesthetic insult to disrupted synaptic proteostasis.

### 3.5 Repeated exposure of neonatal mice to propofol affects their adult emotions, learning, and memory

To assess the long-term behavioral consequences of neonatal propofol exposure, we conducted a series of standardized behavioral tests at postnatal day 35 (P35). P35 was chosen to capture the delayed effects of neonatal propofol exposure. This adolescent stage represents a period of ongoing neural circuit maturation, allowing evaluation of persistent emotional and cognitive impairments. The test battery assessed general locomotor activity, anxiety-like behavior, and cognitive function, including spatial learning and working memory.

**Figure 5:**
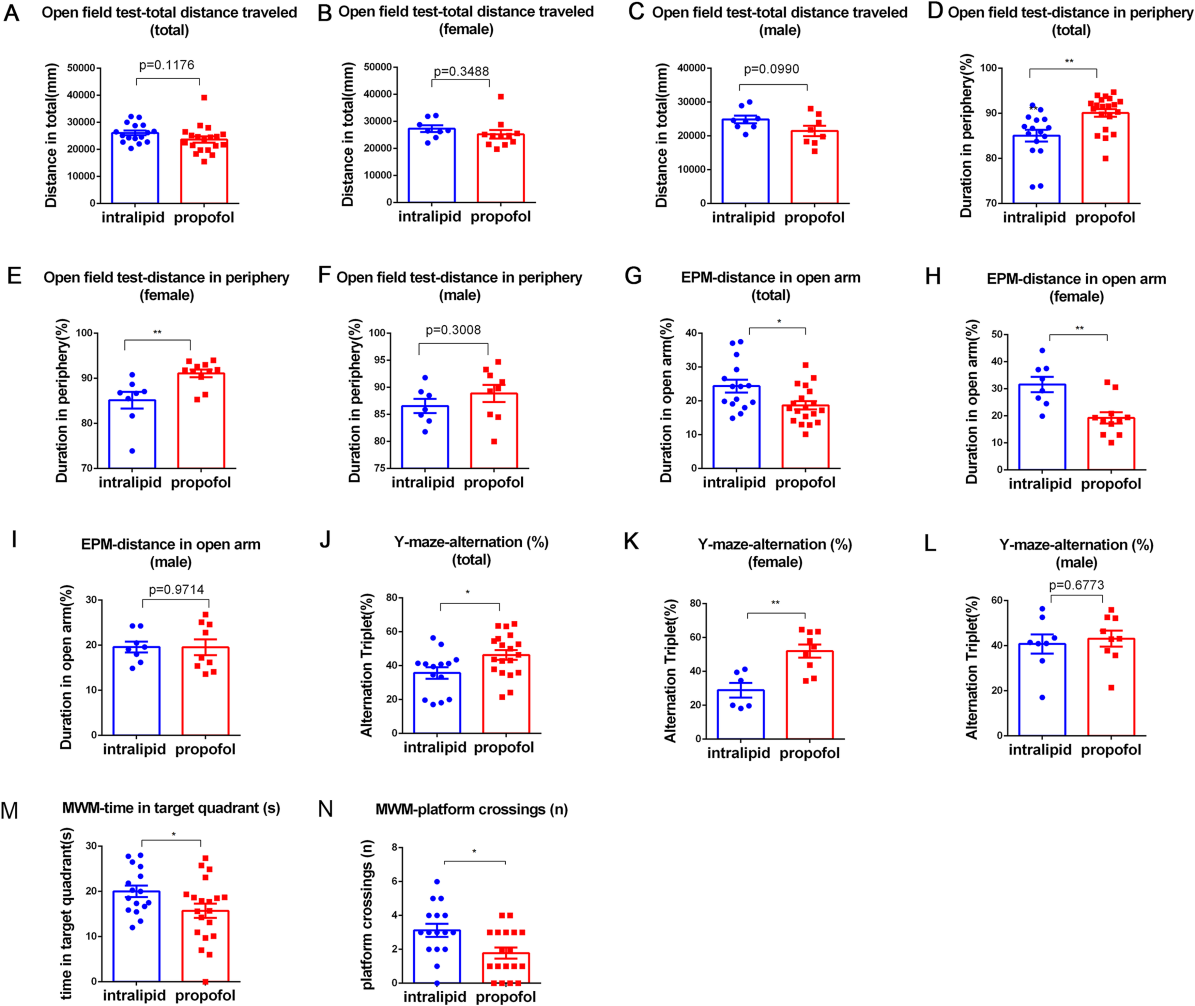
Propofol exposure impairs emotional and cognitive behavior in adolescence. Behavioral assessments were conducted at P35 following neonatal propofol exposure (P5 to P7). **(A–C)** Total distance traveled in the open field test (OFT): **(A)** total, **(B)** female, **(C)** male. **(D–F)** Time spent in the periphery zone of OFT: **(D)** total, **(E)** female, **(F)** male. **(G–I)** Time spent in the open arms of the elevated plus maze (EPM): **(G)** total, **(H)** female, **(I)** male. **(J–L)** Spontaneous alternation percentage in the Y-maze: **(J)** total, **(K)** female, **(L)** male. **(M)** Time spent in the target quadrant during the Morris water maze probe trial. **(N)** Number of platform crossings during the MWM probe trial. Data are expressed as mean ± SEM; *n* = 6–8 per group, *p < 0.05, **p < 0.01, **p < 0.001 *vs*. control group.

In the open-field test, total locomotor distance did not differ significantly between propofol-treated and control mice (Figure 5A), and this was consistent across both females (Figure 5B) and males (Figure 5C), indicating preserved gross motor ability. However, propofol-exposed mice spent significantly more time in the periphery zone (Figure 5D), indicating increased anxiety-like behavior. Sex-stratified analysis revealed that this effect was driven primarily by females (Figure 5E), with no significant difference observed in males (Figure 5F). In the elevated plus maze, although total open-arm time did not differ between groups (Figure 5G), female mice showed a significant reduction in open-arm exploration (Figure 5H), whereas males were unaffected (Figure 5I), further confirming heightened anxiety-like behavior specifically in females. In the Y-maze, propofol-treated mice unexpectedly showed increased total alternation percentage (Figure 5J), an effect driven by females (Figure 5K) but not by males (Figure 5L), suggesting sex-dependent effects on working memory performance.

Spatial learning and long-term memory were evaluated with the Morris water maze. During the training phase, control mice exhibited a progressive decrease in escape latency, whereas the propofol group showed no significant improvement over time, suggesting impaired learning acquisition (Figure 5F). In the probe trial, propofol-treated mice spent significantly less time in the target quadrant (Figure 5M) and completed fewer platform crossings (Figure 5N), indicating long-term memory deficits. Collectively, these findings demonstrate that repeated neonatal exposure to propofol produces persistent disruptions in emotional regulation and cognitive performance during adolescence.

### 3.6 Proposed mechanism: lonidamine intervention partially reverses propofol-induced behavioral deficits

To explore the potential reversibility of propofol-induced behavioral impairments, we tested whether pharmacological modulation with lonidamine, a metabolic inhibitor that influences mitochondrial function and cellular stress signaling (Fantin et al., 2006; Pelicano et al., 2006), could rescue the adverse effects observed in adolescence. Beginning at P30, propofol-exposed mice were randomized to receive either vehicle or lonidamine by intravenous injection every other day until P37, when behavioral assessments were conducted across emotional and cognitive domains.

**Figure 6:**
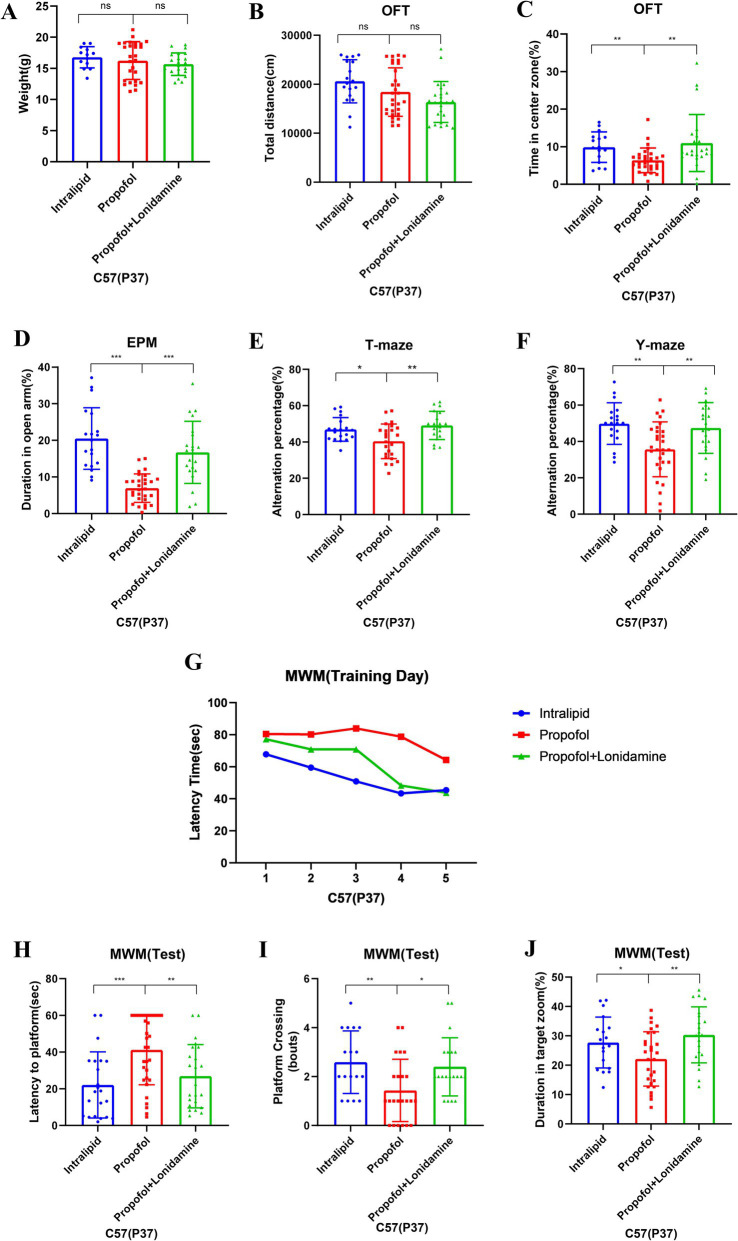
Lonidamine partially rescues propofol-induced behavioral deficits. To assess reversibility, mice exposed to propofol were treated with lonidamine (20 mg/kg, i.v., every other day from P30–P37). Behavioral testing was done at P37. **(A)** Body weight at P37. **(B)** Total distance in OFT (no difference). **(C)** Time in the center of OFT; partially restored by lonidamine. **(D)** Time in the open arms of EPM; increased in the lonidamine group. **(E)** T-maze alternation percentage; improved with lonidamine. **(F)** Y-maze alternation; partially restored. **(G)** Escape latency in balance table hiding task; reduced by lonidamine. **(H)** Time in target zone. **(I)** Latency to target entry. **(J)** Target crossings. Data are mean ± SEM; *n* = 6–8 per group, *p < 0.05, **p < 0.01, ***p < 0.001 vs. control; #*p* < 0.05 vs. propofol group.

As shown in Figure 6A, body weight did not differ significantly among the three groups, indicating that neither propofol nor lonidamine had an effect on general physical development. Locomotor activity, measured as total distance traveled in the open field, was also comparable across groups (Figure 6B). However, propofol-treated mice exhibited increased anxiety-like behavior, spending less time in the center of the open field (Figure 6C), and less time in the open arms of the elevated plus maze (Figure 6D). Notably, lonidamine treatment significantly alleviated both anxiety indicators, partially restoring central exploration and open-arm time.

In the T-maze and Y-maze assessments of working memory, propofol exposure led to a significant decline in alternation performance (Figures 6E,F). Lonidamine administration yielded a partial improvement in alternation rates, suggesting a beneficial effect on spatial working-memory retrieval.

In the Morris water maze, propofol-exposed mice failed to reduce escape latency during training (Figure 6G) and exhibited spatial-memory deficits, including spending less time in the target quadrant (Figure 6H), taking longer to reach the platform for the first time (Figure 6I), and making fewer platform crossings (Figure 6J). Although lonidamine did not fully restore performance to control levels, treated mice showed measurable improvements in all three metrics compared with the untreated propofol group.

It should be noted that the present lonidamine intervention study was designed primarily as a behavioral proof-of-concept. We did not assess synaptic protein expression (e.g., PSD95, SNAP25, BAG3) following lonidamine treatment. Therefore, whether the observed behavioral rescue is mediated by restoration of synaptic protein homeostasis remains to be determined in future studies.

## 4 Discussion

In this observational study, we investigated the role of Bag3 in propofol-induced developmental neurotoxicity by assessing Bag3 expression immediately after exposure (P7) and examining synaptic protein profiles and behavioral outcomes at later stages (P21 and P35-P37). We did not manipulate BAG3 expression, nor did we measure BAG3 and synaptic proteins in the same animals at the same time point. Therefore, causal inference cannot be drawn from the current data. We propose BAG3 as a candidate mediator warranting causal validation, not an established mechanistic link. Our focus on BAG3 is justified by three considerations: (i) its role in synapse-localized autophagy through SYNPO (Ji et al., 2019); (ii) direct regulation of synaptic proteins including PSD95 (Mohseni Ahooyi et al., 2019); and (iii) altered expression after anesthetic exposure in the developing hippocampus (Yu, 2021). Unlike general stress markers such as HSP70 or caspase-3, BAG3 uniquely bridges protein quality control and synapse-specific regulation, making it a compelling candidate to link global cellular stress with localized synaptic dysfunction. These features distinguish BAG3 from general stress markers and position it as a synapse-selective regulator of proteostasis. Based on the temporal sequence observed we propose the following working model: repeated propofol exposure (P5–P7) during the brain growth spurt induces metabolic disturbance (impaired oxidative phosphorylation, calcium dyshomeostasis, enhanced glycolytic flux). In response, the stress-responsive co-chaperone BAG3 is rapidly upregulated in hippocampus and cortex (P7). However, excessive or sustained BAG3 activation may become maladaptive, promoting excessive autophagic turnover of synaptic protein complexes, including PSD95 and SNAP25. This leads to persistent disruption of synaptic protein homeostasis (P21) and excitatory-inhibitory (E/I) imbalance, ultimately manifesting as anxiety-like behaviors and cognitive deficits in spatial learning and working memory (P35–P37). Partial rescue by the metabolic modulator lonidamine supports metabolic stress as an upstream driver. We emphasize that this model is hypothesis-generating; causal validation through BAG3-specific genetic manipulation (e.g., neuron-specific knockout or overexpression) is required before BAG3 can be conclusively designated as a mechanistic hub. Our findings align with recent evidence that neonatal anesthetics disrupt synaptic protein homeostasis (Song et al., 2019) and that sevoflurane upregulates BAG3 leading to dendritic simplification (Zhang et al., 2024). The stress-responsive co-chaperone BAG3 regulates synaptic protein through Hsp70 in various pathological contexts (Behl, 2016; Lin et al., 2022). Extending this to anesthetic neurotoxicity, our findings provide the first evidence that neonatal propofol exposure triggers acute BAG3 upregulation (P7) that temporally precedes and may contribute to subsequent loss of PSD95 and SNAP25 at P21. Complementing our protein-level findings, a recent single-cell transcriptomic study demonstrated that propofol rapidly alters gene expression profiles in human fetal prefrontal cortex neurons, affecting synaptic and stress pathways (Chang et al., 2023). Our data offer unique extensions: (1) a direct temporal link between acute BAG3 upregulation and delayed synaptic loss (at P21); (2) divergent regulation of pre- and postsynaptic markers (GluR1 upregulation coupled with SYN downregulation); and (3) proof-of-concept evidence that postnatal metabolic modulation with lonidamine partially reverses behavioral deficits. Collectively, these findings position BAG3 as a convergent node linking anesthetic-induced metabolic stress to persistent synaptic dysfunction in the developing brain.

Bag3 is a stress-responsive co-chaperone that maintains proteostasis *via* Hsp70-dependent selective autophagy (Gamerdinger et al., 2009; Behl, 2016). In neurons, it supports synaptic maintenance and dendritic integrity (Santoro et al., 2017; Lin et al., 2022), but excessive activity can destabilize synaptic components (Behl, 2016). Our data are consistent with a sequential cascade in which BAG3 upregulation is associated with E/I imbalance and cognitive deficits, suggesting that Bag3 could serve as a candidate convergent effector that might contribute to translating early cellular stress into persistent synaptic dysfunction. Importantly, the temporal precedence of BAG3 upregulation (P7) before synaptic protein loss (P21) is consistent with, though not proof of, a causal role.

A growing body of evidence suggests that metabolic disturbance is a critical in anesthetic-induced neurotoxicity. Previous impair mitochondrial function, disrupt calcium homeostasis, and induce oxidative stress in developing neurons (Jevtovic-Todorovic et al., 2003; Yon et al., 2005). We propose that this metabolic compromise is the primary trigger of Bag3 upregulation. The initial energetic deficit may directly impair energy-intensive processes such as cell proliferation and synaptic maintenance while simultaneously activating Bag3 as a stress-responsive mechanism. Our finding that postnatal intervention with lonidamine partially rescues behavioral deficits further supports this metabolic stress hypothesis. Future studies directly profiling neuronal metabolism (e.g., Seahorse analysis, metabolomics) are warranted to confirm this pathway.

Neonatal propofol exposure induces both structural and functional synaptic alterations. Structurally, PSD95 (involved in synapse stabilization) and SNAP25 (critical for vesicle fusion) were significantly reduced, suggesting impaired synaptic assembly and vesicle cycling (Ehrlich and Malinow, 2004; Südhof and Rothman, 2009). These reductions suggest that propofol impairs synaptic assembly and vesicle cycling during critical windows of development, leading to delayed or incomplete synaptic maturation (Bhattacharyya et al., 2009; Tomasoni et al., 2013). Functionally, we observed a significant upregulation of GluR1 (an AMPA receptor subunit that mediates fast excitatory transmission) along with a marked decrease in SYN (a presynaptic vesicle protein involved in neurotransmitter release), indicating a postsynaptic–presynaptic mismatch consistent with E/I imbalance, a hallmark of neurodevelopmental disorders including autism and schizophrenia (Gao and Penzes, 2015; Sohal and Rubenstein, 2019). This E/I imbalance provides a plausible mechanistic link between the observed molecular changes and the behavioral phenotype. Behaviorally, propofol-treated mice exhibited anxiety-like behaviors and deficits in spatial learning and working memory, aligning with known effects on hippocampus–amygdala and prefrontal circuits (Ming and Song, 2011; Warner et al., 2018).

Postnatal intervention with lonidamine partially rescued propofol-induced behavioral deficits, supporting mitochondrial/metabolic stress pathways as contributors to neurotoxicity. This is consistent with a recent study showing that pharmacological suppression of lactate mitigated postoperative cognitive dysfunction and restored SNAP25 expression (Wang et al., 2025). However, lonidamine is not suitable for pediatric use due to limited blood–brain barrier penetration and systemic toxicity. Future translational efforts should focus on clinically viable strategies such as diazoxide (Bosnjak et al., 2016) or optimized perioperative glycemic control (Feng et al., 2020). Further mechanistic studies are needed to identify downstream effectors of metabolic modulation.

Propofol primarily acts as a positive allosteric modulator of GABAA receptors (Hemmings et al., 2005; Franks, 2008). The persistent dysregulation of synaptic proteins well beyond the period of drug clearance suggests that the initial anesthetic state initiates a secondary cascade of cellular stress responses. We propose that BAG3 upregulation constitutes part of this maladaptive neurodevelopmental remodeling process,distinct from propofol’s canonical acute effects. This temporal dissociation is critical: it implies that targeting downstream stress pathways (e.g., BAG3 or metabolic modulators) may offer a wider therapeutic window than intervening at the receptor level.

Most previous studies focused on apoptotic pathways and oxidative stress (Jevtovic-Todorovic et al., 2003; Yon et al., 2005; Sun et al., 2013). While these mechanisms advanced our understanding of cell death during critical neurodevelopmental windows, they do not fully explain the long-term circuit remodeling or behavioral phenotypes observed in the absence of overt neurodegeneration. Our study shifts focus toward synaptic homeostasis and identifies Bag3 as a novel mediator linking early molecular responses to persistent alterations in synaptic and behavioral dysfunction. To our knowledge, this is the first study to systematically demonstrate that Bag3 upregulation precedes, and potentially mediates, disruption of synaptic proteins after neonatal propofol exposure. Our lonidamine intervention provides proof-of-concept that metabolic regulation may offer a viable therapeutic strategy, expanding the mechanistic framework beyond cell survival to include protein stability and circuit-level functional restoration.

Several limitations should be acknowledged. First and most importantly, the central hypothesis that BAG3 upregulation causes synaptic protein loss remains correlational; we did not manipulate BAG3 expression(knockdown/overexpression). Causal validation is required. Second, we lack electrophysiological assessments [long-term potentiation (LTP), miniature excitatory postsynaptic currents (mEPSCs)] to confirm functional synaptic deficits. Third, while lonidamine partially rescued behavioral deficits, we did not assess its effects on synaptic protein expression. Fourth, the biological sex was not determined, precluding assessment of sex-dependent effects (Gonzales et al., 2015). Fifth, cell proliferation was assessed only at P8 with a short BrdU pulse; long-term survival and differentiation remain unknown. Sixth, the hypothesis that BAG3 upregulation is triggered by metabolic disturbance requires direct validation (e.g., metabolomic or Seahorse analysis). Seventh, Bag3 expression at P21 was not assessed, so the duration of its upregulation is unknown. Eighth, PSD95-SYP colocalization was not performed, limiting direct evidence for synaptic structural disruption. Ninth, Bag3 and synaptic alterations were assessed only at the regional level; cell-type-specific manipulations are needed. Tenth, validation in human-relevant systems (iPSC-derived neurons, brain organoids) would enhance translational relevance. Eleventh, we acknowledge that PSD95-SYP colocalization analysis was not performed in this study. Such analysis would provide direct evidence for disruption of synaptic structural integrity. Future studies should include double immunofluorescence for pre- and postsynaptic markers. These limitations do not invalidate the findings but define clear boundaries for interpretation and guide future studies.

## 5 Conclusion

This study demonstrates that repeated neonatal propofol exposure suppresses hippocampal cell proliferation, acutely upregulates the stress-responsive co-chaperone BAG3, and leads to persistent disruption of synaptic protein homeostasis (e.g., PSD95, SNAP25) along with cognitive deficits in adolescence. By identifying a temporal association in which BAG3 upregulation precedes synaptic protein loss and functional impairment, these findings shift the investigative focus toward BAG3 as a candidate correlative marker and a priority target for future mechanistic studies However, causal validation through genetic manipulation is required before BAG3 can be conclusively designated as a mediator of anesthetic-induced synaptic dysregulation.

## Data Availability

The raw data supporting the conclusions of this article will be made available by the authors, without undue reservation.
